# Supplementary material for: Portable Digital Linear Ion Trap Mass Spectrometer Based on Separate-Region Corona Discharge Ionization Source for On-Site Rapid Detection of Illegal Drugs
Source: Molecules. 2022 May 30;27(11):3506. doi: 10.3390/molecules27113506 (PMC9182377; doi:10.3390/molecules27113506)
Supplement: Supplementary file 1 [file molecules-27-03506-s001.zip › molecules-1732341-supplementary.pdf]

Supporting Information

Portable digital linear ion trap mass spectrometer based on separate-region corona discharge ionization source for on-site rapid detection of illegal drugs

Lingfeng Li [1], Tianyi Zhang [1], Deting Wang [1], Yunjing Zhang [1], Xingli He [1], Xiaozhi Wang [2] and Peng Li [1,*]

1 School of Electronic and Information Engineering, Soochow University, Suzhou 215006, Jiangsu, China.

2 College of Information Science & Electronic Engineering, Zhejiang University, Hangzhou 310027, Zhejiang, China.

* Correspondence: lipengsuda@suda.edu.cn; Tel.: +86-13656249881

Contents

Table S1. Characteristic precursor ions and corresponding product ions of 18 illegal drugs in this study.

Figure S1. Photos of the integrated sample/ionization assembly with the sampler and ion source (a), the ion trap (b), and the whole portable mass spectrometer (c).

Figure S2. Mass spectra of 12 narcotic samples. (a. Methcathinone; b. Ephedrine; c. MDA; d. MDMA; e. Pethidine; f. THC; g. LSD; h. Fentanyl; i. Papaverine; j. 4-FiBF; k. Ocfentanil; l. Sufentanil)

Figure S3. MS/MS spectra of 12 narcotic samples. (a. Methcathinone; b. Ephedrine; c. MDA; d. MDMA; e. Pethidine; f. THC; g. LSD; h. Fentanyl; i. Papaverine; j. 4-FiBF; k. Ocfentanil; l. Sufentanil)

Table S1. Characteristic precursor ions and corresponding product ions of 18 illegal drugs in this study.

Compound	Precursor ion	Detected product ions
1. Methylamphetamine	150.1[M+H]$^+$	119.1 [M-CH_4N]$^+$, 91.1 [M-C_3H_8N]$^+$
2. Methcathinone	164.1[M+H]$^+$	146.1[M-H_2O]$^+$, 131.1[M-H_2O-CH_3]$^+$
3. Ephedrine	166.1[M+H]$^+$	148.1[M-H_2O]$^+$, 133.1[M-H_2O-CH_3]$^+$, 117.1[M-H_2O-C_2H_6]$^+$, 91.1[M-H_2O-C_2H_6-CNH]$^+$
4. MDA	180.1[M+H]$^+$	163.0[M- CH_4N]$^+$, 135.0[CH_2NO]$^+$, 133.0[M-CH_4NO]$^+$, 105.0[M-$C_2H_5NO_2$]$^+$
5. MDMA	194.1[M+H]$^+$	163.0[M-CH_4N]$^+$,
6. Ketamine	238.1[M+H]$^+$	220.1[M-H_2O]$^+$, 207.1 [M-CH_4N]$^+$, 163.0[M-CH_3NH-CH_2OCH_2]$^+$
7. Pethidine	248.2[M+H]$^+$	174.1[M-C_2H_4]$^+$, 220.1[M-$C_3H_6O_2$]$^+$, 202.1[M-$C_3H_6O_2$-H_2O]$^+$
8. Morphine	286.1[M+H]$^+$	268.1[M-H_2O]$^+$, 229.1[M-$CH_3NC_2H_4$]$^+$, 201.1[M-C_4H_7NO]$^+$, 211.1[M-$C_3H_6O_2$]$^+$
9. Cocaine	304.1[M+H]$^+$	182.1 [M-$C_7H_6O_2$]$^+$
10. Tetrahydrocannabinol	315.2[M+H]$^+$	259.2[M-C_4H_9]$^+$, 193.2[M-C_9H_{14}]$^+$, 299.2[M-CH_3]$^+$, 233.2[M-C_6H_9]$^+$, 221.2[M-C_7H_{11}]$^+$, 181.1[M-$C_{10}H_{15}$]$^+$, 135.1[M-$C_{11}H_{17}O_2$]$^+$
11. Lysergic acid diethylamide (LSD)	324.2[M+H]$^+$	223.1[M-$C_2H_5NC_2H_5CO$]$^+$, 251.1[M-$C_2H_5NC_2H_5$]$^+$, 281.2[M-C_2H_5-CH_2]$^+$, 208.1[M-$C_2H_5NC_2H_5COCH_3$]$^+$
12. Fentanyl	337.2[M+H]$^+$	188.1[M-$C_6H_5NHCOC_2H_5$]$^+$
13. Papaverine	340.2[M+H]$^+$	324.1[M-CH_4]$^+$, 202.1[M-$C_2H_6O_2C_6H_4$]$^+$
14. 4-FiBF	369.2[M+H]$^+$	188.1[M-$C_6H_4FNHCOC_3H_7$]$^+$
15. Heroin	370.2[M+H]$^+$	310.1[M-CH_3COOH]$^+$, 328.2[M-CH_3CO]$^+$, 268.1[M-CH_3COO-CH_3CO]$^+$
16. Ocfentanil	371.2[M+H]$^+$	188.1[M-$C_6H_4FNHCOCH_2OCH_3$]$^+$
17. Sufentanil	387.2[M+H]$^+$	238.2[M-$C_2H_5CONHC_6H_5$]$^+$
18. Alfentanil	417.2[M+H]$^+$	268.2[M-$C_6H_5NHCOC_2H_5$]$^+$, 385.2[M-CH_3OH]$^+$

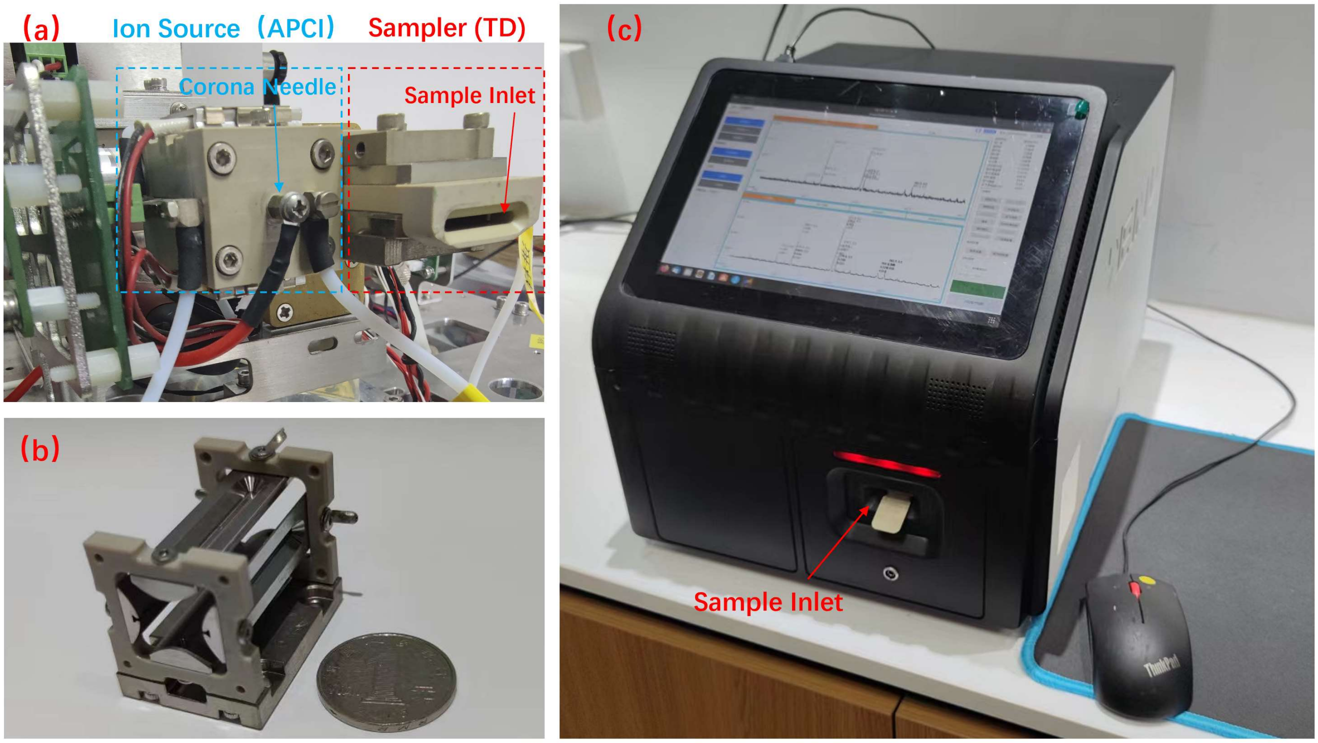

Figure S1. Photos of the integrated sample/ionization assembly with the sampler and ion source (a), the ion trap (b), and the whole portable mass spectrometer (c).

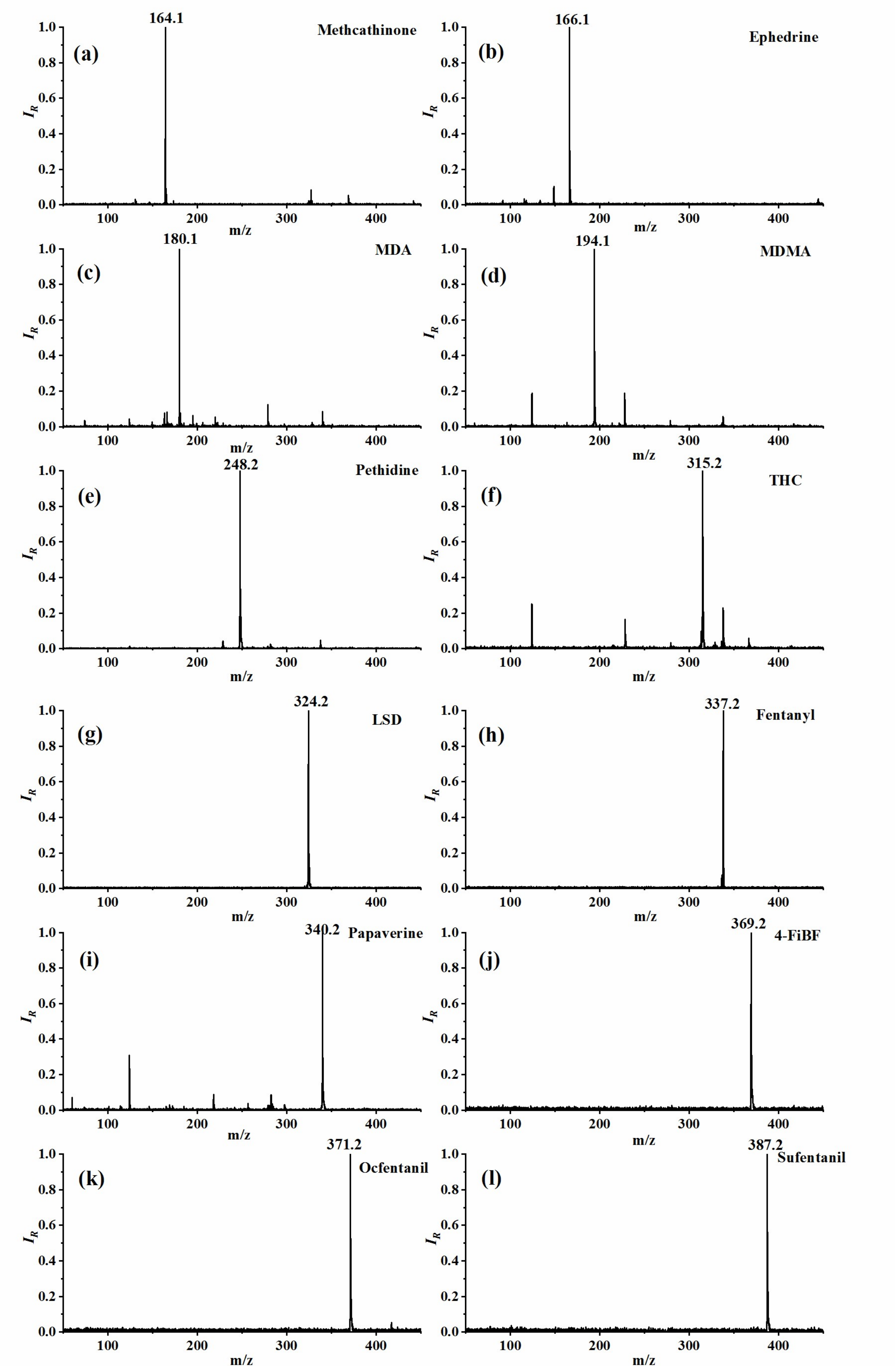

Figure S2. Mass spectra of 12 narcotic samples. (a. Methcathinone; b. Ephedrine; c. MDA; d. MDMA; e. Pethidine; f. THC; g. LSD; h. Fentanyl; i. Papa-verine; j. 4-FiBF; k. Ocfentanil; l. Sufentanil)

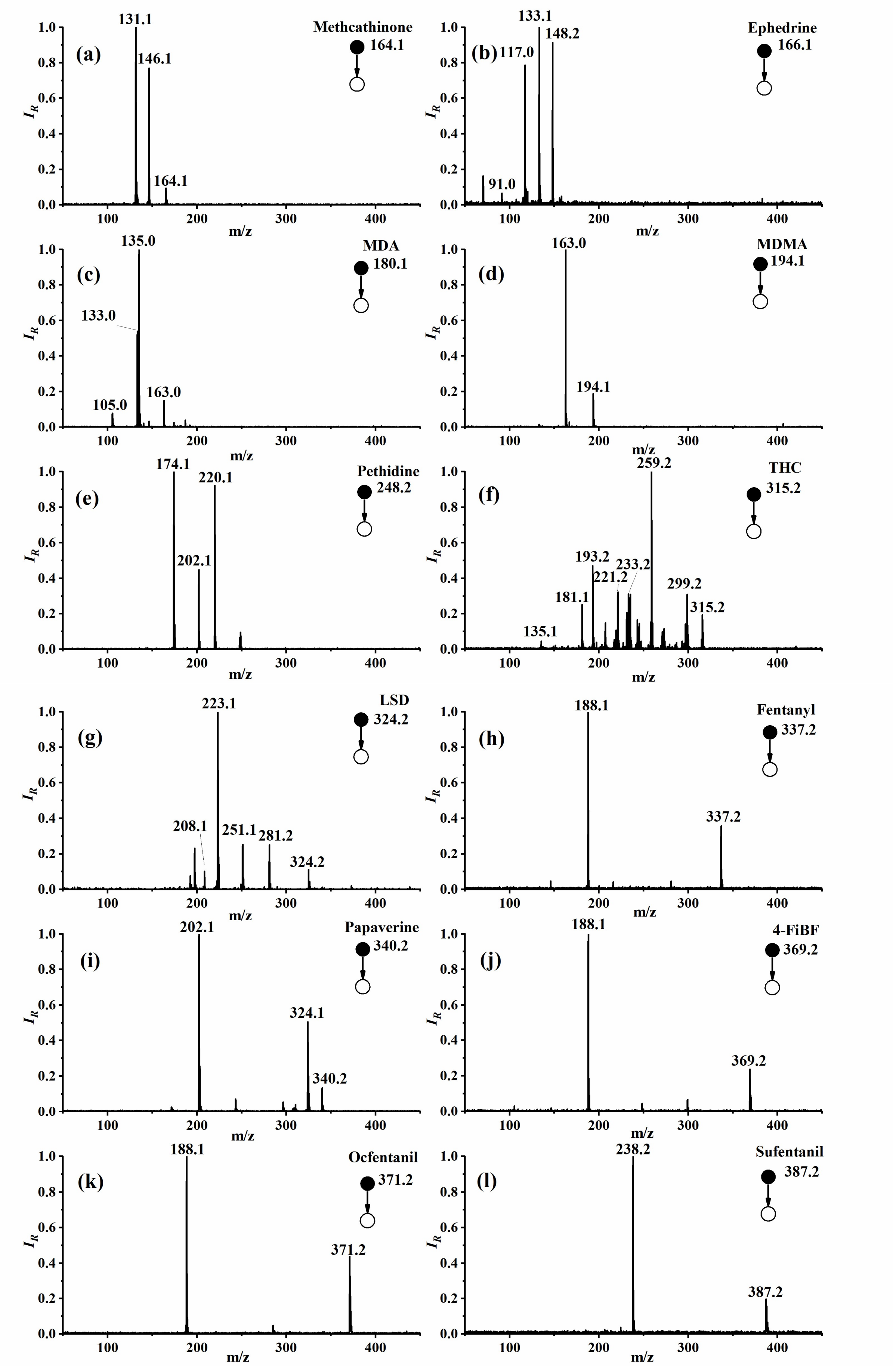

Figure S3. MS/MS spectra of 12 narcotic samples. (a. Methcathinone; b. Ephedrine; c. MDA; d. MDMA; e. Pethidine; f. THC; g. LSD; h. Fentanyl; i. Papa-verine; j. 4-FiBF; k. Ocfentanil; l. Sufentanil)
